# Epidermal growth factor as a potential prognostic and predictive biomarker of response to platinum-based chemotherapy

**DOI:** 10.1371/journal.pone.0252646

**Published:** 2021-06-11

**Authors:** Margot Geens, Sofie Stappers, Heleen Konings, Benedicte Y. De Winter, Pol Specenier, Jan P. Van Meerbeeck, Gert A. Verpooten, Steven Abrams, Annelies Janssens, Marc Peeters, Paul Van de Heyning, Olivier M. Vanderveken, Kristien J. Ledeganck

**Affiliations:** 1 Faculty of Medicine and Health Sciences, University of Antwerp, Antwerp, Belgium; 2 Laboratorium of Experimental Medicine and Pediatrics and Member of the Infla-Med Centre of Excellence, University of Antwerp, Antwerp, Belgium; 3 Department of Gastroenterology and Hepatology, Antwerp University Hospital, Edegem, Belgium; 4 Department of Oncology, Antwerp University Hospital, Edegem, Belgium; 5 Center for Oncological Research, University of Antwerp, Antwerp, Belgium; 6 Department of Pneumology, Antwerp University Hospital, Edegem, Belgium; 7 Global Health Institute, Family Medicine and Population Health, University of Antwerp, Antwerp, Belgium; 8 Data Science Institute, Interuniversity Institute for Biostatistics and Statistical Bioinformatics, UHasselt, Diepenbeek, Belgium; 9 Department of Thoracic Oncology, Antwerp University Hospital, Edegem, Belgium; 10 Department of Oncology, Multidisciplinary Oncological Center Antwerp, Antwerp University Hospital, Edegem, Belgium; 11 Department of Otorhinolaryngology-Head and Neck Surgery, Antwerp University Hospital, Edegem, Belgium; 12 Department of Translational Neurosciences, University of Antwerp, Antwerp, Belgium; Indiana University School of Medicine, UNITED STATES

## Abstract

In this study, we investigated serum epidermal growth factor (EGF) in an oncological population of head- and neck and pulmonary neoplasms and whether serum EGF could serve as a prognostic marker of survival and as a predictive marker for treatment response to platinum-based chemotherapy. A total of 59 oncological patients and a control group of age- and sex-matched healthy volunteers were included in this study. Pre-treatment serum EGF from both groups was determined. Patient’s and tumour characteristics and mortality were recorded during a 5-year follow up period. Baseline serum EGF significantly differed between the oncological patients and the healthy volunteers (p<0.001). Serum EGF was associated with lymph node metastasis (p = 0.004) but not with sex (p = 0.753), age (p = 1.00), TNM stage (p = 0.191) or tumour size (p = 0.077). Neither serum EGF (p = 0.81) nor age (p = 0.55) showed an effect on the patient’s survival. Tumour location was significantly associated with overall 5-year survival (p = 0.003). The predictive capacity of serum EGF of response to chemotherapy was limited (AUC = 0.606), a sensitivity of 80% and a specificity of 56% was observed resulting in a likelihood ratio of a positive and negative test equal to 1.81 and 0.36, respectively. In conclusion, serum EGF levels are 5.5 times higher in an oncological population compared to a control group. Within the oncological population, low serum EGF values are associated with the presence of lymph node metastasis. Further investigation is necessary to determine if the serum EGF levels could serve as a diagnostic biomarker.

## Introduction

Head and neck cancers and lung cancers are highly prevalent tumours with a yearly incidence of 600,000 and 2 million new cases worldwide respectively [1, 2]. The prognosis of both tumour types is poor mainly due to a diagnostic and therapeutic delay. Head and neck neoplasms are frequently diagnosed in an advanced stage [3–12] e. g. the time between malignant transformation and clinical presentation in nose and sinuses malignancies amounts 12 months or more leading to a 20 months median delay in diagnosis [13]. Similarly, two-third of patients with lung tumours are diagnosed in an advanced tumour stage (stages IIIb and IV) [14] with a median delay of 112 days between the first symptoms of lung tumours and the onset of therapy [15].

Besides progress in treatment, early detection of these tumours could play a significant role in improving the prognosis underlining the importance of the discovery of a biomarker for early diagnosis [4, 5, 9, 11, 16]. A large number of potential biomarkers has been investigated for the diagnosis of head and neck neoplasms [17]. However, most of them do not reach the biomarker criteria (high sensitivity, high specificity, early detection of disease, non-invasive detection method, easy to measure, high positive and negative predictive value) to be implemented in clinical practice [17, 18].

Epidermal growth factor (EGF) is a polypeptide consisting of 53 amino acids and is produced at different sites in the body [19, 20]. In humans, EGF is mainly synthetised in the kidneys [20], the digestive tract such as the salivary glands, the oesophageal sub-mucosal glands and the submandibular glands [21]. EGF becomes biologically active after binding to the epidermal growth factor receptor (EGFR) and is involved in the activation of pathways that stimulate cellular proliferation, migration, differentiation and survival in the majority of epithelial tissues, fibroblasts and endothelial cells. Besides, EGF has been shown to be involved in malignant transformation and progression [22–24]. Between seventy-five and seventy-eight percent of the small cell lung tumours overexpress EGF [25]. Also, preclinical studies showed a downregulation of EGF mRNA expression in the rat kidney after treatment with cisplatin [26, 27].

In this study, we aimed to investigate 1) serum EGF in oncological patients with a head and neck or lung carcinoma at the time of diagnosis and 2) whether serum EGF could be a potential biomarker for tumour response to chemotherapy and 5-year survival.

## Materials and methods

### Patients

#### Oncological population

Sixty-two patients were included between 2011 and 2015 at the departments of Otorhinolaryngology, Head and Neck surgery and Medical Oncology of the Antwerp University Hospital (UZA). *Inclusion criteria*: treatment with platinum derivatives. Eligible patients in whom a treatment with platinum derivates was indicated, were consecutively invited to participate in this study. *Exclusion criteria*: diabetes mellitus, an active urinary tract infection, treatment with EGFR-antagonists or calcineurin inhibitors. The present study is part of a larger prospective study for which the in- and exclusion criteria were predefined.

#### Control population

A control group of sixteen age-matched healthy volunteers was recruited at the University of Antwerp (UA) by e-mail advertisement. Each patient confirmed not to be on any medication and completed a questionnaire on medical history and age. Patients on chronic medication or with a chronic illness were excluded.

### Ethical standards

The study is conducted in accordance with the Declaration of Helsinki and principles of Good Clinical Practice. The Ethics Committee of the Antwerp University Hospital has approved the study protocol including patients and healthy subjects (file number 11/5/51) and all patients and healthy controls gave a written informed consent.

### Study design

Preceding the treatment with platinum derivatives, a blood sample was obtained from each oncological patient to determine serum EGF and baseline group characteristics. Relevant clinical data, tumour-related data, risk factors, treatment and the course of disease were collected through the medical records.

From all healthy volunteers, a blood sample was obtained to determine serum EGF levels. Relevant data on the healthy controls were collected through a questionnaire.

### Determination of serum epidermal growth factor

Serum EGF was determined using an EGF Human ELISA kit (Invitrogen, California, USA; product number KHG0061) according to the manufacturer’s guidelines. The ELISA kits with the following lot numbers were used in this study: LOT941600 and LOT622111. The detection limit was 3.9 pg/ml [28].

### Definitions

Therapy response was determined using imaging and clinical features and categorised as complete response, partial response, progressive disease and unknown response. Therapy response was assessed radiographically by experienced clinicians in our center. We dichotomized therapy response in the following manner: a partial and complete response were considered as ‘responsive’ to therapy, this being in contrast to recurrence, a progressive tumour or no response at all. These three findings were classified as ‘non-responsive’ to therapy.

Overall survival was defined as the time from the start of any therapy (chemotherapy, surgery or radiotherapy) until death or until the last follow-up.

### Statistical analysis

Continuous variables are summarized with means ± standard deviations whereas categorical variables are summarized using relative frequencies (proportions) for each of the factor levels. In order to test for differences in means between two groups, an unpaired two-sample Student’s t-test was used upon checking the normality assumption using a Kolmogorov-Smirnov test. Equality of variances in the two groups has been assessed using an F-test. None of the aforementioned assumptions was violated, and solely parametric statistical tests were applied to compare the means in both groups. Two proportions are compared using a Chi-square or Fisher’s exact test, depending on whether all expected cell counts exceeded five. EGF levels were classified as high or low EGF based on a median split method and the association between dichotomized versions of each of the clinical characteristics (e.g., gender, age, etc.) and the EGF serum categorization was assessed using a Chi-square or Fisher’s exact test according to the criterion mentioned above. A Cox proportional hazards model was used to evaluate the impact of age, baseline serum EGF and tumour location on overall 5-year survival. Finally, a ROC analysis was considered to select the optimal cut-off value for EGF, and the predictive performance of the diagnostic test to assess whether therapy with platinum derivatives will be successful based on serum EGF was quantified in terms of the sensitivity and specificity of the test. Next to sensitivity and specificity, we report the likelihood ratio of a positive and negative test, defined as LR+ = sensitivity/(1-specificity) and LR- = (1-sensitivity)/specificity, as test characteristics. All statistical analyses are performed using the statistical software program SPSS (version 25.0). Statistical significance is based on two-sided p-values being smaller than the 5% significance level.

## Results

### Patient characteristics

In total, sixty-two oncological patients with a median age of 61 (25–79) were included in the study of whom 66% was male. They were selected based on their therapy schedule including treatment with platinum derivatives. Tumours were localized at the lung (n = 23), head and neck (n = 36), in the gastrointestinal tract (n = 2) or at an unknown site (n = 1). The latter 3 patients were excluded for further analysis being outnumbered. Since patients with head and neck or lung cancer could differ in parameters such as age, weight, sex, alcohol or smoking habit, these two subgroups were first compared to each other.

### Comparison of lung cancer patients and patients with a head and neck tumour

The baseline characteristics of patients with a lung tumour and patients with a head and neck tumour are summarized in Table 1. The age, baseline EGF, weight, sex, smoking and alcohol habits were comparable between the two groups. Therefore, both groups were taken together for further analysis.

**Table 1:** Comparison of patient and tumour characteristics between lung tumour patients and head and neck tumour patients.

Characteristics		Lung tumour patients (n = 23)	Head and neck tumour patients (n = 36)	p-value
**Demographic data**				
Age (Y)		63.4 ± 6.5	62.9 ± 8.5	0.796
Sex (% male)		57.1	75.8	0.151
Weight (kg)		69.0 ± 12.7	71.6 ± 16.2	0.544
**Laboratory data**				
Baseline serum EGF (pg/ml)		658.6 ± 296.6	655.6 ± 226.5	0.970
**Risk Factors**				
Smoking (%)	Never	19.0	15.2	0.879
	Stopped	47.6	45.5	
	Current	33.3	39.4	
Alcohol (%)	Non-drinker	25	18.8	0.349
	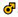 : 1-13U/week, 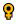 : 1–6 U/week	66.7	46.9	
	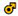 : 13-34U/week, 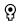 : 7-15U/week	0.0	15.6	
	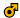 >23U/week, 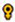 > 15U/week	8.3	18.8	
**Tumour characteristics**				
T stage %T0/%T1/%T2/%T3/%T4/%Tx		4.8/9.5/47.6/14.3/9.5/14.3	9.1/36.4/21.2/18.2/9.1/6.1	0.336
N stage %N0/%N1/%N2/%N3		28.6/0/47.6/23.8	18.2/18.2/57.6/3.0/3.0	0.392
M stage (%)	M0	57.1	93.9	**0.004**
	M1	33.3	3.0	
	Unknown	9.5	3.0	
TNM classification		3.3 ± 1.6	4.5 ± 1.8	**0.001**
**Therapy**				
Intent to treat (%)	Radical	42.9	96.9	**<0.001**
	Palliative	38.1	3.1	
	Unknown	19.0	0.0	
Tumour response (%)	Partial	42.9	18.8	**0.003**
	Complete	23.8	62.5	
	Progressive disease	28.6	3.1	
	Unknown	4.8	15.6	
Nodal response (%)	None	0.0	3.8	**0.001**
	Partial	40.0	15.4	
	Complete	13.3	73.1	
	Progressive disease	33.3	0.0	
	Unknown	13.3	7.7	
1- year overall survival (% Alive)		71.4	93.9	**0.023**
3-year overall survival (% Alive)		38.1	81.8	**0.001**
5-year overall survival (% Alive)		33.3	73.3	**0.028**
Type of chemotherapy (% cisplatinum/%carboplatinum)		57.1/42.9	78.1/21.9	**0.04**

Differences in means between two groups were analysed using an unpaired two-sample Student’s t-test. Two proportions are compared using a Chi-square or Fisher’s exact test, depending on whether all expected cell counts exceeded five. Data are displayed as mean ± standard deviation or percentage. EGF: epidermal growth factor.

The tumour characteristics and survival rate were significantly different between lung tumour patients and head and neck tumour patients.

### EGF in a control versus an oncological population

Five patients below 50 years were excluded for the data analysis on EGF as a predictive marker of therapeutic response and patient’s survival. Our research group previously demonstrated in healthy subjects that the serum EGF value decreases with age until the age of 50 while it remains stable from 50 years on [28]. Indeed, the serum EGF concentration was significantly higher in the oncological patients below 50 years when compared to older patients (p = 0.009), who were therefore excluded to avoid an age-based bias.

The age-matched control population (n = 16) had a median age of 54.9 (50.9–74.2) years. Thirty-one percent was male and 69% was female.

**Fig 1:**
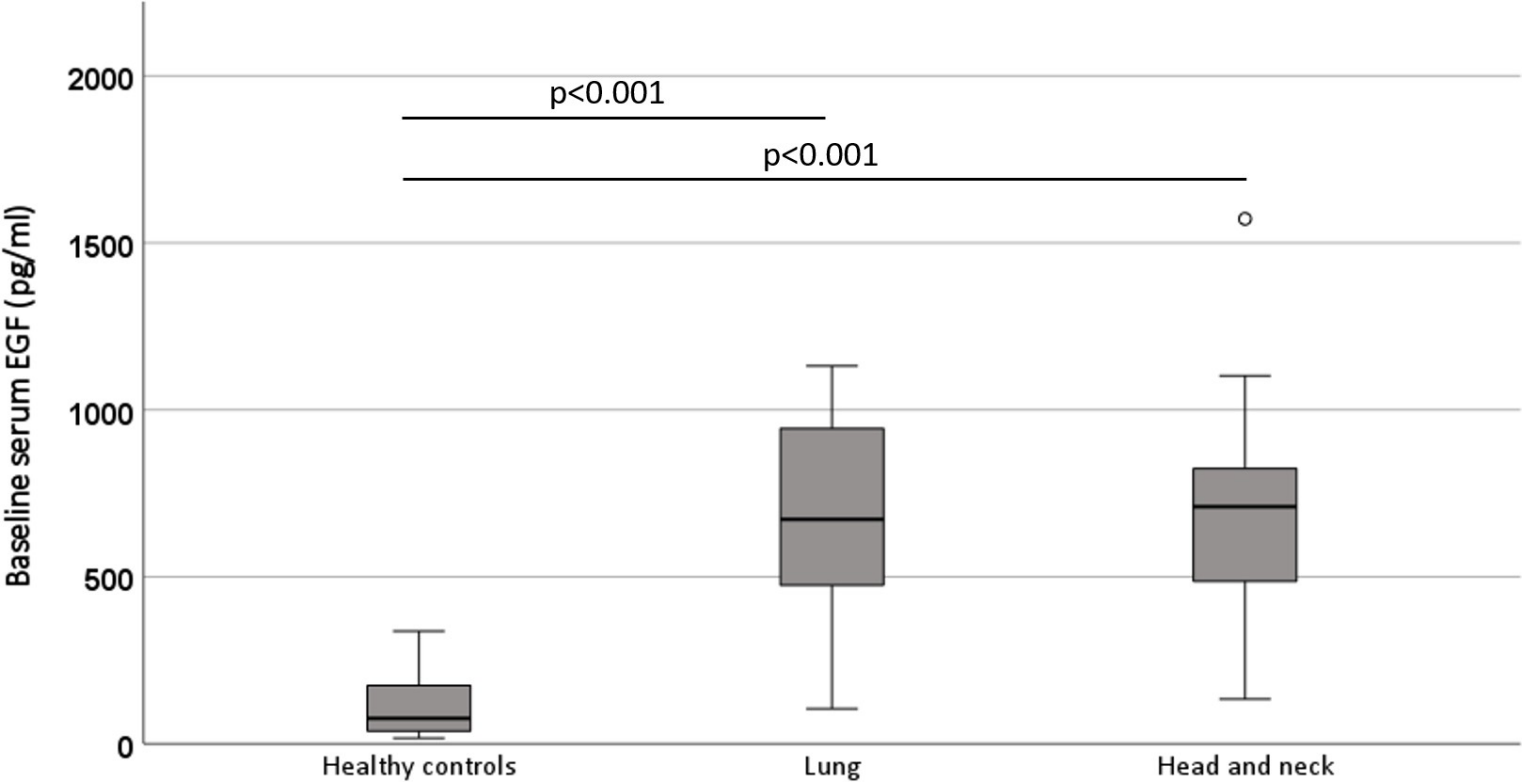
Simple boxplot of baseline serum EGF in healthy controls, patients with lung cancer and patients with head and neck cancer. The baseline serum EGF levels in both oncological populations were significantly higher compared to the healthy controls (p<0.001). EGF: epidermal growth factor.

Baseline serum EGF levels significantly differed between the control and oncological groups (p<0.001). The baseline serum EGF level in the healthy volunteers was 120.26 ± 109.31 pg/ml, compared to 656.68 ± 249.79 pg/ml in the oncological patients. When re-analysing the data by separation of both tumour location subgroups, EGF did not differ between patients with head and neck cancer when compared to patients with lung cancer (p = 1.000) while both groups had significantly higher serum EGF when compared to healthy subjects (p<0.001; Fig 1).

### Relationship between serum EGF concentrations and clinical features

The oncological group was next divided into two groups: low EGF (<660.7 pg/ml) and high EGF (≥660.7 pg/ml) levels according to the median baseline EGF level. The association analysis showed a relationship between EGF levels and lymph node metastasis (p = 0.004). Although non-significant given the limited sample sizes, there seems to be some evidence for an association between serum EGF and tumour size (p = 0.077). No association was found for EGF level by sex, age and TNM stage (see Table 2).

**Table 2:** The association between serum EGF levels and clinical characteristics of lung- and head and neck tumour patients.

Characteristics		Total number	Low EGF levels	High EGF levels	P value
Sex	Male	31	15	16	0.753
	Female	15	8	7	
Age (Y)	<55	8	3	5	1.000
	≥55	38	20	18	
Tumour size	<3cm	16	5	11	**0.077**
	≥3cm	16	10	6	
TNM stage	I-II-III	14	9	5	0.191
	IV	28	12	16	
Lymph node metastasis	No	11	1	10	**0.004**
	Yes	35	22	13	

The association between serum EGF levels and clinical characteristics of lung- and head and neck tumour patients was determined using a Chi-square test and a Fisher’s exact test depending on whether all expected cell counts exceeded five.

### EGF as a prognostic marker for overall survival

The impact of age, tumor location and serum EGF on overall survival of oncological patients was investigated using a Cox regression model. As shown in Table 3, in our population, neither serum EGF (p = 0.81) nor age (p = 0.55) showed an effect on the patient’s survival. In contrast, tumour location was significantly associated with overall 5-year survival (p = 0.003).

**Table 3:** The impact of age, tumour location and serum EGF on overall survival of oncological patients.

				95,0% CI for Exp(B)	
Variable	B	p-value	Exp(B)	Lower	Upper
Baseline serum EGF	0.000	0.810	1.000	0.998	1.002
Tumour location	1.464	**0.003**	4.321	1.660	11.252
Age (years)	0.022	0.546	1.022	0.952	1.097

The Cox regression model showed that nor serum EGF nor age were associated with the patient’s survival while tumour location had a significant effect. EGF: epidermal growth factor.

**Fig 2:**
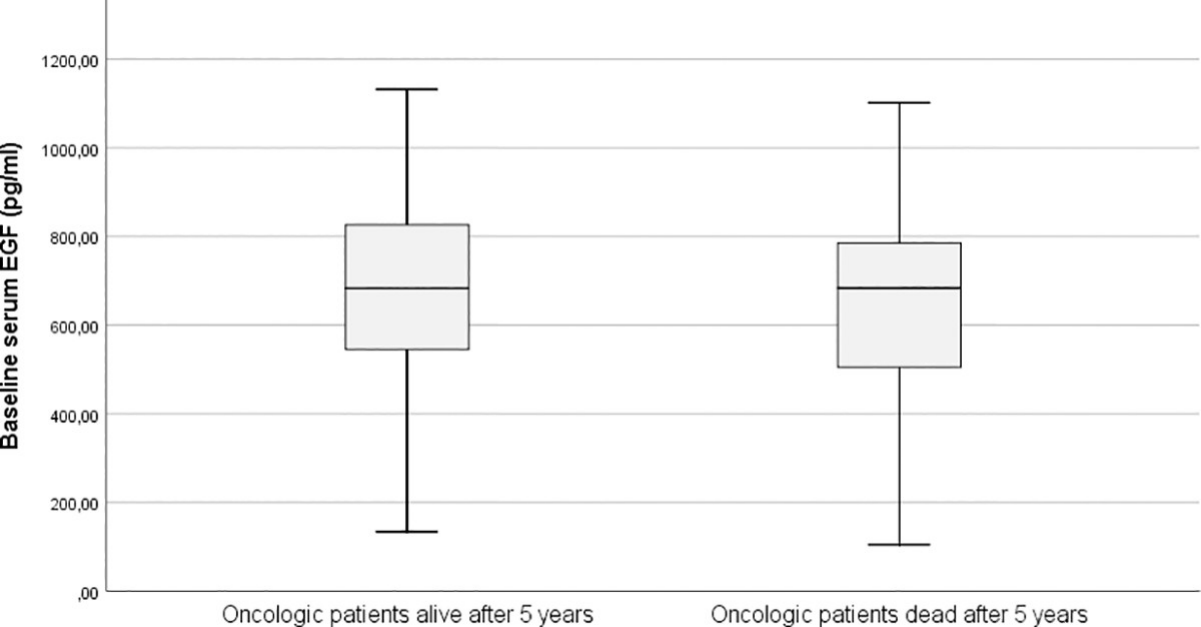
Simple boxplot of baseline serum EGF levels in oncological patients according to 5-year overall survival. The baseline serum EGF levels were similar in the oncological population who was alive after 5 years and the oncologic population who was dead after 5 years (P = 0.868).

Serum EGF did not significantly differ between oncological patients who survived 5 years after start of therapy (685.84 ± 243.74 pg/ml) and patients who died in the first 5 years after start of therapy (672.50 ± 249.70 pg/ml; p = 0.868) (Fig 2).

### EGF as a predictive marker for therapy response

**Fig 3:**
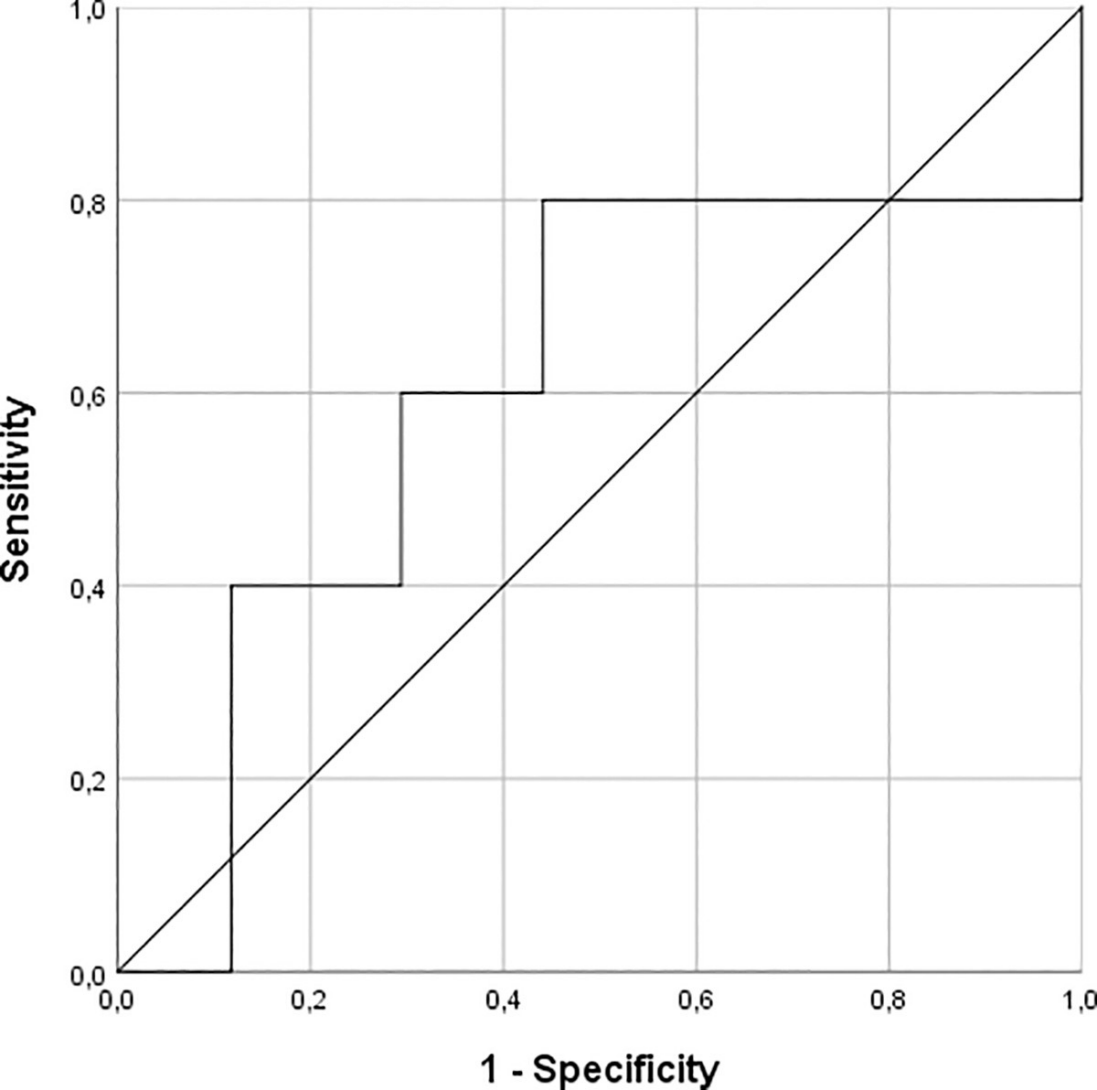
ROC curve of EGF as a predictive marker of platinum-based. Roc analysis shows a low accuracy of EGF as a predictive biomarker of therapy with platinum derivatives within the oncological population (AUC = 0.606).

EGF has a rather low predictive capacity for the response to therapy with platinum derivatives with an area under the curve of 0.606 (Fig 3). At a serum EGF level of 700 pg/ml, a sensitivity of 0.80 and a specificity of 0.56 was observed resulting in a likelihood ratio of a positive and negative test equal to 1.81 and 0.36, respectively.

## Discussion

In this study, serum epidermal growth factor (EGF) as a biomarker in patients with head and neck tumours and lung tumours, was investigated. Our results showed that baseline serum EGF differed significantly between healthy controls and the oncological population with 5.5-fold higher EGF levels in the oncological population. Serum EGF was significantly associated with the presence of lymph node metastasis and a trend to significance was found between serum EGF and tumour size. The 5-year survival rate was not significantly associated with baseline serum EGF. EGF has a rather low predictive capacity for the response to therapy with platinum derivatives with an area under the curve of 0.606, a sensitivity of 80% and a specificity of 56%.

Several studies have examined the correlation between EGF receptor (EGFR) expression and tumour stage in patients with head and neck squamous cell carcinoma (HNSCC), all concluding that the expression of EGFR is heterogeneous and that the expression of this receptor is independent from tumour stage [29–32]. EGF, in contrast, has been shown to be involved in malignant transformation and progression [22–24]. Our study showed a significant difference in baseline serum EGF levels between healthy controls and an oncological population, while no difference was observed between patients with lung cancer versus head and neck cancer. A significant increase in serum EGF levels in patients with oral squamous cell carcinoma (OSCC) [33], papillary thyroid cancer patients [34] and in lung tumour patients [35] has been described earlier. In the latter population, serum EGF levels were statistically distinguishable from healthy controls at advanced TNM stages (III-IV) as well as the early tumour TNM stages (I-II) with the highest EGF levels in the early tumour stages [35]. From our results and other studies, it seems that EGF predominantly plays a role in tumour progression in the early phases, when the tumour is smaller and before lymph nodes are affected. Remarkably, Lemos-Gonzalez *et al*. found significant lower serum EGF levels in lung tumour and head and neck tumour patients compared to healthy controls. However, they included serum samples from healthy subjects via the blood transfusion centre in Galicia [36] but did not report the age of the healthy donors. We recently published reference serum EGF concentrations per age and clearly demonstrated that serum EGF decreases with age [28]. An age discrepancy between oncological patients and the healthy subjects might thus explain the reported lower serum EGF levels in the oncological patients, whose mean age was above 60 years. Besides EGF, six other ligands are involved in the activation of the EGFR, thereby inducing specific cellular responses and intracellular trafficking events. It would be interesting if these EGFR ligands such as TGF-alpha, amphiregulin or epiregulin, might serve as biomarker in head and neck or lung cancer patients as we found for EGF. Literature study showed that TGF-alpha expression levels at the tumour level are increased in e.g. pancreatic cancer [37] and gastrointestinal cancer [38]. With respect to head and neck cancer, higher TGF-alpha expression at the tumour level was associated with a worse outcome [39]. Lemos-Gonzalez *et al*. did not find any significant differences in serum levels between healthy subjects and patients with head and neck cancer nor for TGF-alpha nor for amphiregulin [36]. However, as mentioned before, this study used serum from healthy blood donors as a control samples so an age difference between healthy subjects and HNC patients might interfere with the results.

Serum EGF was significantly associated with lymph node metastasis. No association was found with sex, age and TNM stage and also the association with the tumour burden did not reach significance. Lin *et al*. demonstrated in a population of 152 patients a significant correlation between the serum EGF levels and lymph node metastasis (p = 0.026), worse survival (p = 0.002) and advanced TNM stage (p = 0.040) in oral squamous cell carcinoma patients (OSCC). No association was found with sex and age [33], which is comparable to the results of our study. There are, however, some differences between our study and the Lin study. Our study population consisted of fewer patients with tumour TNM stage I-II-III (n = 14) in comparison with *Lin et al*. (n = 87), making our conclusion on the association between EGF levels and TNM stage less robust. Also Konturek *et al*. showed a positive association between the EGF levels and the pTNM papillary thyroid cancer stage [34]. The association between the serum EGF levels and lymph node metastasis or TNM stages as shown by us and others is strengthened by a preclinical *in vitro* study of Ohnishi *et al*. who illustrated that EGF elevates the invasion activity of an HSC3 oral cancer cell line. EGF also increases matrix metalloproteinase 9 (MMP9) activity, which plays a role in cancer progression, cancer invasion and metastasis [40].

In contradiction to our results, lung tumour patients showed a significant association of serum EGF with sex (p = 0.004) and smoking status (p = 0.011). No correlation between serum EGF levels and age was found for lung tumour patients [35], which is in line with our data.

The survival rate decreased with higher serum EGF levels—although not statistically significant. Besides, no significant difference was found between the mean serum EGF levels of patients who survived five years after start of therapy and the levels of those who died in the first five years after start of therapy. Accordingly, from our results, it appears that baseline serum EGF levels cannot serve as a prognostic marker for survival. Other studies, however, did prove the significance of serum EGF levels as a prognostic marker of survival. Lin *et al*. found that in OSCC patients, higher preoperative serum EGF levels were associated with a significantly poorer survival [33]. Therefore, a large prospective study in a well-defined oncological population would be useful to distinguish whether serum EGF levels could serve as a prognostic biomarker for survival.

In our study, EGF had a low accuracy (AUC = 0.606) as a predictive marker for the sensitivity of therapy with platinum derivatives within the lung tumour population and the head and neck tumour population. Neither likelihood ratio for a positive (1.81) nor a negative (0.36) serum EGF test for therapy response was acceptable for the purpose of a predictive biomarker. Although uncertainty around the estimates quantifying the predictive performance of the test based on serum EGF levels (e.g., AUC) could be added by bootstrapping the observed data non-parametrically, the serum EGF level solely seems to lead to an inadequately high discrimination between therapy success or not. Therefore, we postpone a more thorough investigation of the predictive ability given a newly defined prediction rule. in which other relevant biomarkers can be potentially added to the serum EGF level, to future work. To our knowledge, no other data have been published on serum EGF levels as a predictive marker for treatment response.

Due to the fact that this study was part of a larger prospective trial, patients treated with EGFR antagonists were excluded from the study. Patients who express high levels of EGFR or patients who express mutants of EGFR might therefore be missed. Nevertheless, it seems plausible that EGF could have a biological role in the tumor growth, and therefore subsequent studies should include patients treated with EGFR antagonists as well. Another limitation of this study is the relatively low number of included patients which limits the interpretation of the results. However, even in this small population, we were able to show important data with an increased baseline serum EGF in oncological patients compared to healthy subjects and the association of EGF with the presence of lymph nodes. Another advantage of our study also was the inclusion of an age and sex matched control population consisting of healthy volunteers. Our results should encourage other research groups to further investigate the potential of EGF as a diagnostic marker. With respect to this, the relation between EGF and other tumor characteristics are worth further investigation including parameters such as human papillomavirus status, histological features, tumor type or tumor grade. Particularly in lung cancer, it would be interesting to see if EGF levels vary between non-small cell lung cancer versus small cell lung cancer.

## Conclusion

In conclusion, baseline serum EGF was 5.5-fold higher in an oncological population when compared to healthy controls. The capacity of serum EGF to predict the 5-year overall survival or response to a chemotherapeutic treatment with platinum derivatives is rather limited. Further research is needed to determine whether baseline serum EGF levels could serve as a diagnostic biomarker in head and neck or lung cancer patients.
